# MCM2 and Carbonic Anhydrase 9 Are Novel Potential Targets for Neuroblastoma Pharmacological Treatment

**DOI:** 10.3390/biomedicines8110471

**Published:** 2020-11-03

**Authors:** Patrizia Garbati, Raffaella Barbieri, Davide Cangelosi, Carlo Zanon, Delfina Costa, Alessandra Eva, Stefano Thellung, Matilde Calderoni, Francesca Baldini, Gian Paolo Tonini, Paola Modesto, Tullio Florio, Aldo Pagano

**Affiliations:** 1 IRCCS Ospedale Policlinico San Martino, 16132 Genova, Italy; p.r.garbati@gmail.com (P.G.); raffaella.barbieri@edu.unige.it (R.B.); delfina.costa@hsanmartino.it (D.C.); tullio.florio@unige.it (T.F.); 2 Laboratory of Molecular Biology, IRCCS Giannina Gaslini Institute, 16147 Genova, Italy; davidecangelosi@gaslini.org (D.C.); alessandraeva@gaslini.org (A.E.); 3 Istituto di Ricerca Pediatrica (IRP)—Fondazione Città della Speranza, Haematology-Oncology Lab, 35127 Padova, Italy; c.zanon@irpcds.org (C.Z.); gp.tonini@irpcds.org (G.P.T.); 4 Department of Internal Medicine and Centre of Excellence for Biomedical Research, University of Genova, 16132 Genova, Italy; stefano.thellung@unige.it; 5 Department of Experimental Medicine, University of Genova, 16132 Genova, Italy; calderoni.matilde@gmail.com (M.C.); baldinifrancesca92@gmail.com (F.B.); 6 National Reference Center for Veterinary and Comparative Oncology-Veterinary Medical Research Institute for Piemonte, Liguria and Valle d’Aosta, 10154 Torino, Italy; Paola.Modesto@izsto.it

**Keywords:** neuroblastoma, carbonic anhydrase 9, MCM2, ciprofloxacin, acetazolamide

## Abstract

To overcome the lack of effective pharmacological treatments for high-risk neuroblastoma (HR-NB), the development of novel in vitro and in vivo models that better recapitulate the disease is required. Here, we used an in vitro multiclonal cell model encompassing NB cell differentiation stages, to identify potential novel pharmacological targets. This model allowed us to identify, by low-density RT-PCR arrays, two gene sets, one over-expressed during NB cell differentiation, and the other up-regulated in more malignant cells. Challenging two HR-NB gene expression datasets, we found that these two gene sets are related to high and low survival, respectively. Using mouse NB cisplatin-treated xenografts, we identified two genes within the list associated to the malignant stage (MCM2 and carbonic anhydrase 9), whose expression is positively correlated with tumor growth. Thus, we tested their pharmacological targeting as potential therapeutic strategy. We measured mice survival and tumor growth rate after xenografts of human NB treated with cisplatin in the presence of MCM2/carbonic anhydrase 9 inhibitors (ciprofloxacin and acetazolamide). MCM2 or carbonic anhydrase 9 inhibition significantly increased cisplatin activity, supporting their possible testing for NB therapy.

## 1. Introduction

In order to investigate neuroblastoma (NB) cell differentiation, we recently developed a novel in vitro model based on the controlled expression of NDM29 non-coding (nc)RNA in SKNBE-2 cell line [1].

NDM29 RNA is part of a set of ncRNAs transcribed by RNA Polymerase III, recently isolated and studied as pivotal regulators of different key processes in nervous system cells [2,3,4,5,6]. In particular, NDM29 is located in the first intron of the ASCL3 (Achaete Scute-like homologue 3) gene; its over-expression promotes differentiation of NB cells into cells with neuron-like phenotype and causes inhibition of NB development and malignancy [1,7,8]. It also leads to reduced expression of multidrug resistance proteins (MDRs) in malignant NB cells, thus increasing susceptibility to cytotoxic drugs commonly used in NB therapy [1,9].

This NB model is composed by four SKNBE-2 clones genetically engineered to integrate extra copies of NDM29 ncRNA transcriptional unit. According to the different numbers of extra copies integrated and the different efficiency of expression (in turn, dependent on the integration sites of the extra copies) these clones express NDM29 RNA at different levels which are directly correlated with the levels of differentiation/maturation toward a neural-like phenotype. SKNBE2 cells permanently transfected with the empty vector (pMock), express similar NDM29 ncRNA levels than wt cells, whereas NDM29-overexpressing cells, show a ncRNA expression increase of approximately 2-fold (S2 clone), 5.4-fold (S1 clone) and 8.4-fold (S1.1 clone) as compared to pMock cells. S1.1 cell phenotype was extensively characterized in terms of morphology, physiology, and tumorigenic potential, showing properties of functional neurons able to self-organize a neural network that spontaneously exhibits physiological spiking and bursting activities [8]. Since the progressive differentiation toward mature neurons occurring across the different stage/clones of the NB model is accompanied by a proportional loss of the tumorigenic and malignant potential, this model recapitulates a multistep/multiclone cell reinstruction model of NB differentiation that spans from the highly malignant to the non-malignant stage [1,4,7,8,10]. We also demonstrated that the induction of differentiation commitment in the context of anticancer therapy is possible by the pharmacological induction of NDM29 ncRNA expression which, in turn, increase the efficacy of the anticancer treatments. In particular, NDM29 expression drives the downregulation of the multidrug resistance proteins (MDRs) reverting tumor refractoriness to chemotherapeutics [9].

Taking advantage of this information, recently, we obtained in preclinical studies a significant enhancement of the survival of NB-xenografted mice treated with cisplatin in combination with the pharmacological induction of NDM29 expression [9].

These encouraging results prompted us to pursue the possible identification of novel targets for NB treatment in order to identify more specific and efficacious drugs for the treatment of high-risk (HR)-NB.

In this study, we used the NDM29-based model to identify genes which are progressively up-regulated proceeding from the non-malignant stage of mature neuron-like cells to the fully malignant stage, and those up-regulated during cell differentiation exhibiting the highest expression level in the differentiated non-malignant cells. In our experimental setting, these two gene lists represent pro-malignant and pro-differentiation sets where the pro-malignant genes might represent novel targets for innovative NB therapies. We also demonstrate that these genes trace a genetic signature of NB representing significant prognostic predictors.

We also report that, in a subcutaneous xenotransplant NB model the growth index of NB tumor nodules, the rate of GD2 positive malignant cells and the cell proliferation index are highly correlated with the expression level of two genes of our malignant set, MCM2 and carbonic anhydrase 9 (CA9), validating these genes as possible novel targets for NB therapy. Indeed, we here show that, in vivo, the pharmacological inhibition of MCM2 and/or CA9 is efficacious to inhibit/block tumor growth, thus representing a novel promising therapeutic strategy.

Altogether these results show that NDM29-dependent multistep NB differentiation model recapitulates several features of NB cell differentiation and is suitable to identify novel prognostic genes and discover specific pharmacological inhibitors of their products, thus providing novel potential therapeutic molecules to be further investigated in preclinical studies.

## 2. Experimental Section

### 2.1. Gene Expression Datasets

Two publicly available datasets have been used for the gene expression analysis. The first dataset, that we referred as RNAseq498 [11], contains the gene expression profile of 498 neuroblastomas measured by the Illumina HiSeq 2000 RNAseq platform (GSE62564). The second one, that we referred as Versteeg88 [12], contains the gene expression profile of 88 tumors measured by the Affymetrix Human Genome U133 Plus 2.0 platform (GSE16476).

### 2.2. Mice

Homozygous male and female NOD-SCID mice were purchased from the breeding of Animal Facility of the IRCCS Ospedale Policlinico San Martino (Genova, Italy), where animal housing was carried out. Mice were kept under standard laboratory condition: 22–24 °C with 12 h light/12 h dark cycle. Standard mouse feed and water were provided ad libitum. During the experimental procedure, efforts were made to minimize animal stress or discomfort. Mice were used in experiments at about 8 weeks of age. Animal care and experimental procedures were approved by the Italian Ministry of Health, as well as by the Institutional Ethics Committee of the IRCCS Ospedale Policlinico San Martino, and performed in accordance with the guidelines of the European Community for the use and care of live animals used in scientific experimentation.

### 2.3. In Vivo Tumor Formation

NOD/SCID mice were subcutaneously injected with a cell suspension of SKNBE2 wt cells (3 × 106 cells) in serum-free RPMI. To monitor tumor development at injection sites, mice were observed every two days. Subsequently, tumor-bearing mice were randomly divided into two groups: (I) untreated mice (*n* = 7 mice) and (II) cisplatin (3 mg/kg/dose)-treated group (*n* = 6 mice). Treated group received weekly intraperitoneal drug administration. Cisplatin administration, as well as the measurement of the tumor masses, was started when the subcutaneous tumor bulk reached a threshold diameter of 5 mm. Tumor size was measured every two day using a digital caliper and the volume calculated using the following formula: length^2^ × width × π/6. At the maximum tumor volume of 2.2 cm^3^, mice were euthanized by CO_2_ narcosis. Tumor growth rate was calculated by the formula: final volume (approximately 2.2 cm^3^) minus the initial volume (when tumor mass reached the threshold diameter of 5 mm)/number of days to reach the maximum tumor volume. Experimental procedures involving animals were carried out in accordance with the guidelines of the European Community for the use and care of live animals, approved by the Italian Ministry of Health (D.lgs.vo 116/92) and by the Ethics Committee of the Animal Facility of IRCCS Policlinico San Martino (protocol DGSAF 0001448-A). Efforts were made to minimize animal stress/discomfort.

### 2.4. Immunohistochemistry

After mouse euthanasia, tumor mass was surgically removed and fixed in 4% neutral-buffered formalin, dehydrated, and embedded in paraffin using standard histologic techniques. Sections (5 µm thick) were cut, and immunohistochemistry was performed. Antigen retrieval was carried out in citrate buffer (pH 6) for 9 min in microwave oven. Hydrogen peroxide 3.6% for 10 min was employed on the tissue slices to eliminate the endogenous peroxidase activity.

To perform the immunohistochemical staining, tissue sections were incubated in presence of the following antibodies: mouse anti-CA9 (NBP1-51691, Novus biological, Milano, Italy) 1:400 dilution; mouse anti-MCM2 (Thermo scientific, Milano, Italy) 1:500; or rabbit anti-Ki67 (NB600-1252 Novus biological, Milano, Italy) 1:200 concentration. The reactivity of tissue towards GD2 was assessed by using a portion of the tumor frozen embedded in OCT (Killik, Bio-Optica, Milano, Italy) cooled by liquid nitrogen and cut into 5 mm sections by a cryostat at −20 °C. Sections were allowed to dry for 24 h and shortly fixed on paraformaldehyde vapor for 5 min. The sections were then stained using mouse anti-human disialoganglioside GD2 monoclonal antibody (MAB2052 Millipore, Temecula, CA, USA) 1:200 dilution. As negative controls, primary antibodies were omitted. The immunoreactions ware visualized using 3,3′-diaminobenzidine (DAB) chromogen (Dako—Glostrup, Denmark). Slices were counterstained using Harris hematoxylin (Applichem Panreac, Darmstadt, Germany). TUNEL assay was performed using the in situ cell death detection kit, AP (Roche Diagnostic GmbH, Mannheim, Germany) following the manufacturer’s instructions. After this reaction, the chromogen used was Fast-Red Substrate System (Thermo Scientific, Fremont, CA 94539, USA) following manufacturer’s instructions. Tissue slides were scanned using Aperio AT2—Scanner (Leica Microsystems Srl, Via E. Bugatti 12, Milano, Italy) at ×20 magnification. After getting eight images per group randomly, the positive area in slices was quantified using ImageJ software downloaded from the NIH website (http://rsb.info.nih.gov/ij).

### 2.5. Immunofluorescence Detection

Cells were fixed using 4% formaldehyde diluted in in 0.1 M phosphate-buffered saline, washed and then blocked with 5% normal goat serum (Merck Millipore, Darmstadt, Germany) containing 0.1 % Triton X-100 in PBS). Cells were then incubated overnight at 4 °C in blocking solution with primary antibodies: mouse anti-nestin, clone 10C2 1:200 (MAB5326, Merck Millipore, Darmstadt, Germany), mouse anti-tubulin β 3 (TUB β3) 1:1000 (Biolegend, San Diego, CA, USA), rabbit anti-MAP2 1:500 (ab75059, Abcam, Cambridge, UK), rabbit anti-doublecortin 1:500 (ab18723, Abcam, Cambridge, UK). Secondary antibodies Alexa Fluor 568 goat anti-rabbit IgG and Alexa Fluor 647 goat anti-mouse IgG (Molecular Probes, Milano, Italy) were used at 1:500. Nucleus was stained with Hoechst 33342 (GeneCopoeiaTM, Rockville, MD 20850, USA). Images were captured using a Zeiss Axiovert 200-M inverted microscope equipped with ApoTome slide module (Zeiss, Oberkochen, Germania), through a ×63 objective, and processed by using Zeiss AxioVision 4.8 software (Zeiss, Oberkochen, Germania).

The resulting images were processed, analyzed using ImageJ. Fluorescence intensity was measured by the integrated density of the area of selected cell to which it was subtracted mean fluorescence of background readings.

### 2.6. Cell Cultures

Mock, S2, S1, and S1.1 cells, obtained as described elsewhere [1,8], were grown in RPMI 1640 medium (EUROCLONE S.p.A. Pero, Milano, Italy), 10% FBS (EUROCLONE S.p.A. Pero, Milano, Italy), L-glutamine (2 mM; EUROCLONE S.p.A. Pero, Milano, Italy), penicillin–streptomycin (100 U/mL/100 ug/mL; EUROCLONE S.p.A. Pero, Milano, Italy) (standard medium), geneticin G418 (Roche, Milano, Italy) 200 μg/mL. Moreover, S1.1 medium, when cells reached 50% confluence, was enriched with trans-retinoic acid 10µM (Sigma Aldrich, Milano, Italy) and 0.1 μL/mL puromycin (Sigma Aldrich, Milano, Italy). After 5 days in presence of trans-retinoic acid, 50 ng/mL, recombinant human β-BDNF (PeproTech, London, UK) and 10 ng/mL recombinant human β-NGF (PeproTech, London, UK) were added.

A certificate confirming that all SKNBE2 human cell lines have been authenticated using STR (or SNP) profiling within the last three years has been included.

### 2.7. Methylcellulose Colony Formation Assay

Clonogenic assays were performed using a methylcellulose medium consisting of RPMI with 0.4% methylcellulose (Methocult H4100, StemCell Technologies, Vancouver, BC, Canada), 10% fetal bovine serum, 100 U/mL penicillin/streptomycin, and 2 mM L-glutamine. Mock, S2, S1, and S1.1 cells were plated at a density of 500 cells in methylcellulose medium in humidified 6-well plates. Colonies were counted 12 days after plating. Images were captured at 20× or 4× magnification on an EVOS FL digital inverted microscope (Advanced Microscopy Group, WA, USA). Data were obtained from three independent assays performed in duplicate. Each data represents an average of eight microscope fields for each cell line.

### 2.8. Quantitative Real-Time RT-PCR Analysis

Total RNAs from samples were extracted using TRIzol reagent (Invitrogen, Carlsbad, CA, USA) according to the manufacturer’s protocol and subjected to reverse transcription by Transcriptor First Strand cDNA Synthesis Kit (Roche, Milano, Italy), with random hexamer as primers. A low-density PCR array (RT2 Profiler PCR Array, Cat. No. 330231, Qiagen, Hilden, Germany) was used to examine the expression patterns of 96 genes involved in cancer pathways, following manufacturer’s instructions. To confirm the results obtained by Qiagen array, the total RNA from samples was measured by real-time quantitative RT-PCR using PE ABI PRISM@ 7500 Sequence Detection System (Perkin Elmer Corp./Applied Biosystems, Foster City, CA, USA) and Sybr Green method following manufacturer’s instructions. No-template control tubes (NTC), containing water instead of template mRNA, were also run under the same conditions for each gene. Relative transcript levels were determined from the relative standard curve constructed from stock cDNA dilutions and divided by the target quantity of the calibrator following manufacturer’s instructions. The primers used are reported in Supplementary Material Online 1.

### 2.9. Statistical Analysis

A one-way ANOVA followed by post-hoc Tukey HSD (Honestly Significant Difference) Test was used to determine significance between the analyzed groups (*p* values < 0.05) with a 95% confidence interval (http://astatsa.com/OneWay_Anova_with_TukeyHSD/). The data are presented as average ± SD. On graphs, statistical significance is displayed as *p* < 0.05 (one asterisk), *p* < 0.01 (two asterisks).

### 2.10. Bioinformatic Procedures and Statistical Analysis

We used log2-transformed gene expression values. We selected gene symbol as reference annotation. In the Affymetrix platform the highest probe set expression was associated to the gene symbol [12]. Heat map visualization and unsupervised k-means clustering used Morpheus: versatile matrix visualization and analysis software (https://software.broadinstitute.org/morpheus).

Univariate analysis with Cox proportional regression model evaluated the prognostic value of the genes, taken singularly. Overall survival curves have been plotted by Kaplan Meier method and compared by the log-rank test. Survival analyses used survival package [13]. A Package for Survival Analysis in S. version 2.38, https://CRAN.R-project.org/package=survival [14] and GraphPad Prism version 6.0 for MAC, GraphPad Software, San Diego California USA, www.graphpad.com. *p* Values lower than 0.05 were considered significant.

## 3. Results

### 3.1. NDM29-Driven Neuroectodermal Differentiation Delineates a Bidirectional/Four Clones Model of Neuroblastoma Progression

We developed a novel genetically engineered in vitro model of NB including four cell clones encompassing different tumorigenic potentials. The clones progressively span from the malignant mycN-amplified SKNBE2 cells to post-mitotic non-malignant neuron-like cells. All the clones were originated from the same cells by NDM29 transfection so that their phenotypic differences are ascribable only to the level of NDM29 over-expression and to the differentiation that it drives. We obtained three clones in which NDM29 was overexpressed by about 2-fold in S2 clone, 5.4-fold in S1 clone, and 8.4-fold in S1.1 clone, as compared to mock transfected SKNBE2 cells.

**Figure 1:**
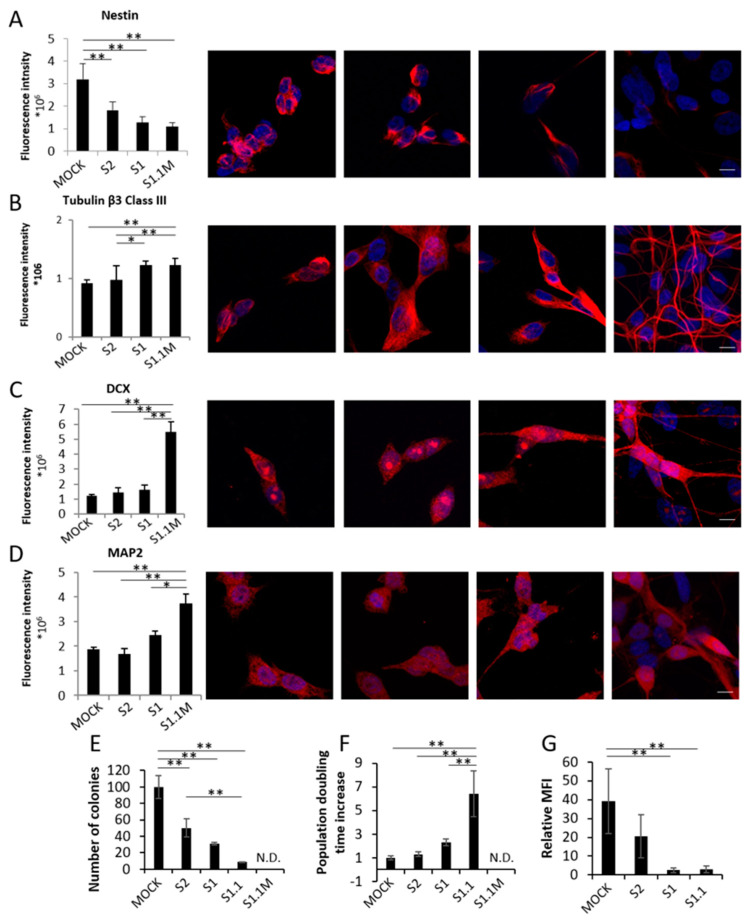
NDM29-driven differentiation of neuroblastoma cells The expression of neuron-specific protein markers in NDM29-overexpressing cells was evaluated by IF: (**A**) Nestin, NES; (**B**) Tubulin β3 Class III; TUBβ3, (**C**) Doublecortin, DCX and (**D**) Microtubule Associated Protein 2, MAP2 were detected and their relative fluorescence intensity was calculated (see Material and Methods Section), scale bars 20 µm; (**E**) Decrease of colony forming showing the progressive lack of malignancy from Mock to S 1.1 cells cultured in the presence of morphogens. Statistical analysis was performed excluding S1.1M cells that did not form colonies in methylcellulose; (**F**) The increase of population doubling-time correlates to the increase of NDM29 expression level. Statistical analysis was performed excluding S1.1M cells because of their fully mature, non-proliferating stage; (**G**) FACS analysis of GD2^+^ cells in different cell populations shows a linear reduction of GD2^+^ cells in clones overexpressing NDM29 ncRNA. Statistical significance is displayed as *p* < 0.05 (*), *p* < 0.01 (**).

The procedures used to engineer Mock, S2 and S1 has been already reported [1] whereas the process to generate S1.1 is reported in [8]. In order to assess the progressive acquisition of neuronal traits during NB differentiation from Mock to S1.1 cells, we first measured in the four clones by the means of IF intensity signal the expression of four markers of neural differentiation: nestin (NES), Tubulin β3 Class III (TUBβ3), Doublecortin (DCX), and Microtubule Associated Protein 2 (MAP2). As shown in Figure 1A the stem cell-related marker NES is progressively downregulated in cells with increased NDM29 expression, whereas TUBβ3, DCX and MAP2 are all up-regulated, as it occurs during cell differentiation. These data confirm that NDM29 expression levels correlate with a progressive acquisition of a differentiated neuron phenotype across the different clones (Figure 1A–D).

The progressive loss of malignancy of NDM29 high expressing cells was then demonstrated by the decrease of colony-forming ability that showed a continuous decrease from Mock cells (taken as 100% malignant) to 50% of S2, 31% of S1, 8.5% of S1.1, and the inability to form colonies in methylcellulose of S1.1 cells cultured in the presence of morphogens (Figure 1E).

Furthermore, population doubling time of the different clones supports their progressive differentiation reaching a maximal increase of 70.9 fold in S1.1 cells, and a post-mitotic non-proliferating stage when the cells were cultured in the presence of morphogens and fully matured to neurons (Figure 1F). Indeed, although S1.1 cells are endowed with all the excitatory properties of the neuron [7,8] they reach the most mature phenotype (self-organizing functional neural networks that autonomously spikes and bursts) when cultured in the presence of brain derived neurotrophic factor (BDNF), nerve growth factor (NGF), and retinoic acid (RA) [8].

Next, we analyzed by FACS the possible alteration of the rate of GD2+ cells in the different cell populations. Indeed, GD2 disialoganglioside expression is restricted to tumors of neuroectodermal origin, such as neuroblastoma, glioma, and melanoma [15,16]. Results showed a dramatic linear reduction of GD2+ cells in clones progressing toward differentiation with a maximal inhibition in S1 and S1.1 cell populations (6 and 7% of the mock signal, respectively) according to the progressive loss of malignancy along the differentiation in this model (Figure 1G).

### 3.2. Two Defined Subsets of Genes Are Associated with Tumorigenesis or Neurogenesis, Respectively, in the NB Models

Since the different grades of differentiation, malignancy and the phenotypic traits of the multistage NB model recapitulate neural differentiation in one direction and the acquisition of malignancy in the opposite direction, we used this model to identify genes that are progressively regulated across differentiation or during the acquisition of malignancy, thus putatively involved in carcinogenesis. To this aim we measured the expression change in the different clones of about 80 cancer-related genes taking advantage of Real Time Low Density Arrays. Notably, in this gene expression analysis, we considered only genes whose expression modulation was progressive across the four stages of differentiation. This selection was intended to isolate novel candidates that play pivotal roles in NB malignancy.

**Figure 2:**
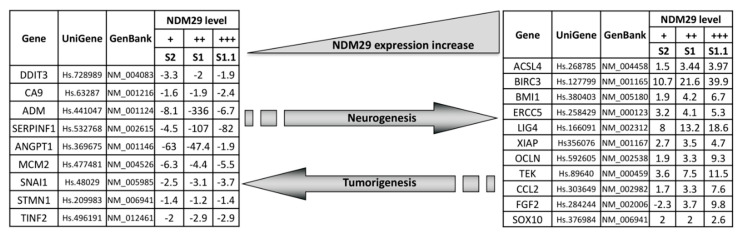
Different expression of genes associated with tumorigenesis or neurogenesis.

This preliminary analysis provided us two lists of 9 and 11 genes [hereafter referred to as “progression of Tumorigenesis Genes” (PTGs) and “Progression of Neurogenesis Genes” (PNGs) respectively] grouped on the basis of their putative active role in differentiation or carcinogenesis (Figure 2). The expression modulation of PTGs and PNGs was then tested and confirmed by specific real-time RT-PCR determinations (Supplementary Material Online 2).

The first analysis of these two gene sets suggested a preliminary interpretation of the possible role played by single genes: BMI1, ERCC5, and LIG 4 are key actors in DNA damage repair systems, and are highly expressed in non-malignant S1.1 cells whereas they are downregulated in Mock cells, in which, as a consequence, a genome instability and a significant increase of the mutational rate is expected. In this scenario also the overexpression of DDIT3 in the malignant stage is in agreement with a strong response to a DNA damage increase [17]. CA9 gene, abundantly expressed in malignant cells, participates to the hypoxic condition response, typical of several solid tumors [18,19], whereas SERP1F, ANGPT1, and STMN1 may cooperate in the inhibition of angiogenesis [20,21,22]. SNAIL participates to the maintenance of an undifferentiated state by inhibiting the expression of ectodermal differentiation genes [23]. Last, MCM2, a key player in DNA replication, is over-expressed in highly proliferating undifferentiated cancer cells [24].

### 3.3. PTGs and PNGs Are Prognostic and Stratify NB Patients in Two Groups

**Figure 3:**
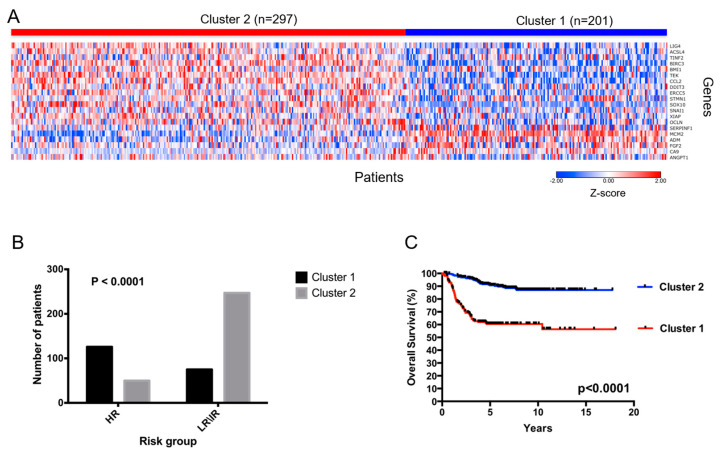
Progression of Tumorigenesis Genes (PTGs) and Progression of Neurogenesis Genes (PNGs) are prognostic and stratify neuroblastoma (NB) patients in two groups. (**A**) The panel shows a heat map for the 20 genes in the 498 neuroblastoma tumor dataset. The expression for each gene has been scaled using a z-score transformation and is represented by pseudo-colors in the heat map. Red color corresponds to high level of expression and blue color corresponds to low level of expression. The color key is located in the bottom right side of the plot. The 498 patients (columns) were divided into two groups by unsupervised k-means clustering. Cluster 1 consists of 201 patients and cluster 2 consists of 297 patients. The expression values of the 20 genes are shown (rows). The name of the genes is reported on the right side of the heat map. (**B**) Panel shows the number of patients of each cluster for each risk group. Risk was divided in two groups on the basis of the prognosis. The groups are high-risk vs. low-\intermediate- risk. Association between the cluster and the risk group was assessed by Fisher exact test. Fisher *p* value is reported in the top left part of the plot. *p* value lower than 0.05 is considered significant. (**C**) Panel shows the Kaplan Meier for 498 neuroblastoma patients divided in two clusters. Curves are relative to the patient overall survival expressed in years. Blue and red curves represent good and poor prognosis patients, respectively. The *p*-value of the log-rank test is shown.

To assess the prognostic value of PTGs and PNGs, we split the publicly available gene expression dataset of 498 NB patients into two groups by applying an unsupervised k-means clustering to the gene expression values of the 20 genes (Figure 3A). This analysis stratified patients into two clusters including 201 (cluster 1) and 297 (cluster 2) cases, respectively. Gene expression was clearly different between the two clusters of patients as it is shown in the heat-map in Figure 3A. We then associated the gene expression characteristics of the patients within the clusters and the patients’ risk groups. High-risk NB patients who are often associated with a poor prognosis and outcome composed the first group and intermediate- or low-risk patients who are often associated with a good prognosis and outcome composed the second one. Fisher’s exact test was used to evaluate the statistical significance of this association. Analysis showed a significant association between the two classifications, as shown by the number of patients of each cluster in the two risk groups (*p* < 0.0001, Figure 3B). In detail high risk NB patients mainly segregate within cluster 1 and intermediate- low-risk in cluster 2. These findings prompted us to compare survival outcome between the two clusters of patients. This was analyzed by Kaplan Meier curves and log-rank test for the overall survival (Figure 3C). Analysis showed a significant separation between the two survival curves suggesting that the expression of the 20 genes identified as PTGs and PNGs take part in the definition of the NB aggressiveness.

### 3.4. The Overexpression of MCM2 Gene is Strongly Associated to Low Patients’ Survival

We then assessed whether the expression of specific PTGs and PNGs may better correlate to NB aggressiveness. To this end, we performed a univariate survival analysis using the two publicly available gene expression datasets relative to 498 and 88 NB patients. We found that MCM2, CA9, SERPINF1, SOX10, OCLN, ACSL4, XIAP, and ERCC5 expression display a significant prognostic value as far as overall survival (*p* < 0.05, Table 1). A higher expression of MCM2, CA9, and SERP INF1 was associated with a higher risk of succumbing to the disease (HR > 1, Table 1). Whereas higher expression of SOX10, OCLN, ACSL4, XIAP, and ERCC5 was associated with a lower risk of death from the tumor (HR < 1, Table 1). This analysis highlighted that MCM2 is the most significant gene among the PTGs and PNGs in both datasets (*p* < 0.0001, Table 1). Although it is known that all members of the mini-chromosome maintenance complex are up-regulated by MYCN in NB [25], our results demonstrate that MCM2 gene is an unfavorable prognostic factor for NB patients and is the most relevant prognostic gene among both PTGs and PNGs.

### 3.5. Gene Ontology Analysis Shows a Strong Involvement of MCM2 in Replicative Stress

**Figure 4:**
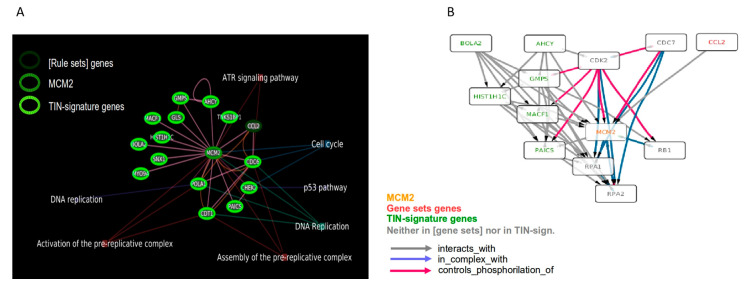
Gene interaction analyses. (**A**) The panel shows the MCM2 direct interactors identified by the EGAN software using KEGG, NCI-Nature Pathway Interaction Database, Panther, and Reactome as connections sources; the most relevant enriched pathways are also shown. Most of the interactors are listed in the Transcriptional Instability (TIN)-signature genes. (**B**) Pathway Commons gene interactions ‘core’ derived from the genes shortlisted in panel A. The more interconnected genes are shown revealing, besides (rule sets) and TIN-signature genes, other interactors including RPA1 and RPA2.

In a previous work we noticed that MCM2 ranked within the top 1% of Transcriptional Instability (TIN)-related genes [26] so we further investigated the resulting pathway relationships and implications by performing a pathway analysis on both rule sets gene lists and the TIN-signature gene list [26]. We first identified the direct MCM2 interactors within the genes belonging to the rule sets list and to the TIN-signature using the EGAN software [27], which integrates information from a number of different pathway databases such as KEGG, NCI-Nature Pathway Interaction Database, and Reactome, among others (Figure 4A). This selected shortlist of 16 genes, including MCM2, was then used to identify an extended list of directly interacting genes using the Pathway Commons database of gene interactions (version 7) [28]. The resulting enlarged net of interactions was eventually pruned of the genes with the lowest number of connections thus, leaving a “core” of thirteen highly interconnected “hub” genes including MCM2, one Rule Sets gene, six TIN-signature genes and five interactors not included in the above mentioned lists (Figure 4B). This central group of genes confirms the strong involvement of MCM2 in the replication stress response including two known effectors RPA1 and RPA2 (Figure 4B).

### 3.6. Growth Rate of NB Mass Correlates with the Expression of MCM2 and CA9 Genes in a Mouse In Vivo Model

In order to preliminarily assess whether PTGs are suitable novel targets for innovative NB therapies, we selected two of them to whether their pharmacological inhibition interferes with NB growth in a pilot experiment using a mouse model of NB xenograft.

Considering that (1) the high significance of MCM2 gene expression in survival prediction, (2) the overexpression of MCM2 (and of the whole MCM family) is induced by Myc-N, (3) small molecule inhibitors of MCM2 (ciprofloxacin, lovastatin, trichostatin A, widdrol) are available and already FDA-approved for use in the treatment of other diseases, and (4) the key role played by MCM2 overexpression in the transcriptional instability typical of NB cells [26], we chose MCM2 as candidate for a possible target therapy in a mouse model of NB in vivo.

The second gene chosen as experimental target was CA9, a gene already considered a promising target in breast [29,30,31], lung [32,33], colon [34,35], and bladder [36] carcinomas, and in medulloblastomas and glioblastomas [37,38,39]. Indeed, membrane localization of CA9, which allows its targeting by both small molecule inhibitors and monoclonal antibodies, together with the availability of specific FDA-approved inhibitors [40], strengthened our choice to consider this protein as a valuable target for a possible novel NB therapy.

First, in order to assess whether a correlation between the expression of MCM2/CA9 genes and the growth rate of the tumor mass exists, we took advantage of a mouse model of subcutaneously injected NB tumor xenograft, using SKNBE2 cells. Two experimental groups of mice (*n* = 4/group) were analyzed: (a) untreated and (b) weekly treated with cisplatin (5 mg/kg/dose), thus recapitulating the nodule growth delay expected by chemotherapy and its possible effects on tumor progression.

After about 7–14 days all the mice developed a subcutaneous cancer nodule whose growth rate (mm^3^/die) was determined by daily measuring the volume of each nodule. Once the nodules reached the established tumor mass threshold (2.2 cm^3^) the individuals were sacrificed, and the nodules analyzed by immunohistochemistry staining with mouse anti-MCM2 and mouse anti-CA9 antibodies. The rate of positivity of each nodule was obtained by the means of percentage of signal positivity of the total area quantitatively analyzed by Image J software (see Materials and Methods Section). The same analysis was performed in order to determine (1) the amount apoptotic cells (positivity to Tunel reaction), (2) proliferation rate (Ki67 staining) and (3) malignant potential (positivity to Anti-GD2 antibody), in order to correlate the expression level of MCM2 and CA9 not only to the growth of the tumor but also to its expected dangerousness.

**Figure 5:**
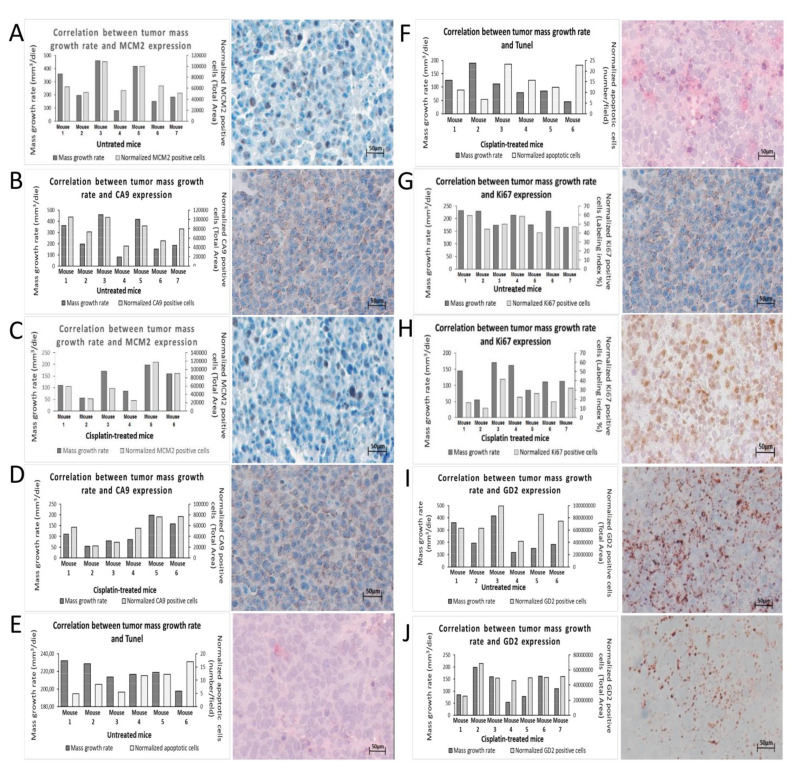
Immunohistochemical analysis of tumor nodule. (**A**) Correlation between tumor masses growth rates and: CA9 in untreated and (**C**) cisplatin-treated mice, (**B**) MCM2 in untreated and (**D**) cisplatin-treated mice. (**E**) Correlation between tumor masses growth rates and apoptosis in untreated and (**F**) cisplatin-treated mice. (**G**) Correlation between tumor masses growth rates and Ki67 in untreated (**H**) and cisplatin-treated mice. (**I**) Correlation between tumor masses growth rates and GD2 in untreated and (**J**) cisplatin-treated mice. The graphs show in left y-axis the growth rate of the tumor masses, expressed as mm^3^/day, and in the right y-axis the positive area of staining calculated by ImageJ software. On the x-axis, the mice involved in the study.

Next, the growth rate of the nodules of all the individuals of each experimental group was averaged and statistically correlated to the rate of positivity of the nodules to each specific marker. Results showed that the average of tumor growth rate of untreated mice strongly correlates to the expression level of both MCM2 and Ca9 genes (Pearson’s correlation values 0.8397 (*t* test 0.0002) and 0.8841 (*t* test 0.0001), respectively) (Figure 5A,B). Similarly, we also found a strong Pearson’s correlation between the expression of MCM2/CA9 and tumor nodule growth (Pearson’s correlation values 0.8557 (*t* test 0.0078) and 0.9010 (*t* test 0.0025), respectively) in the group of mice treated with cisplatin, thus strengthening what observed in untreated mice (Figure 5C,D).

Since the positivity to Tunel reaction (apoptosis), Ki67 (proliferation), and GD2 (malignant potential) are canonically considered for the evaluation of tumor dangerousness, we tested their correlation with tumor nodules growth and compared it with the same parameter obtained from MCM2 and CA9 in order to further clarify their key role in NB progression. As expected, we found that there is a strong negative correlation between apoptosis and tumor growth rate in both untreated and treated mice (Pearson’s correlation values −0.757 (*t* test 0.0001) and −0.705 (*t* test 0.001), respectively) (Figure 5E,F). Furthermore, a positive correlation between tumor nodule growth and Ki67 signal in both untreated and treated mice (Pearson’s correlation values 0.39 (*t* test 0.0001) and 0.53 (*t* test 0.0001), respectively) was observed, although its extent was moderate indicating that Ki67 is less sensitive than MCM2 and CA9 (both strongly correlated to tumor growth index) in the prognosis of tumor progression (Figure 5G,H). Similarly, the correlation between GD2 expression and tumor growth rate was moderate in both the groups of mice [Pearson’s correlation values 0.530 (*t* test 0.0001) and 0.673 (*t* test 0.0001) respectively] showing that in our experimental system also GD2 is less prognostic of tumor aggressiveness than MCM2 and CA9 (Figure 5I,J). These results are summarized in Supplementary Material Online 3.

Altogether these results show that both MCM2 and CA9 expression in NB nodules are strongly correlated with the growth rate of tumor masses and negatively correlated to their apoptotic rate, suggesting the possible use of these two proteins as both prognostic elements and possible pharmacological targets for NB innovative therapies should be pursued.

### 3.7. The Pharmacological Inhibition of MCM2 and CA9 Potentiates the Effects of Cisplatin Therapy In Vivo

In light of the above results, we assessed the therapeutic relevance of the pharmacological inhibition of CA9 and MCM2 activities, using two FDA-approved membrane-permeable small molecule inhibitors, the mild diuretic acetazolamide (5-acetamido-1,3,4-thiadiazole-2-sulfonamide) and the antibacterial agent ciprofloxacin (1-cyclopropyl-6-fluoro-4-oxo-7-(1-piperazinyl)-1,4-dihydro-3-quinolinecarboxylic acid) [41,42]. We performed an in vivo pilot experiment to assess whether treatment with these two drugs might exert an inhibitory effect on NB tumor nodule growth and/or increase the overall survival of treated mice. Acetazolamide and ciprofloxacin were administered alone or in co-treatment with cisplatin in order to test also possible beneficial effects through synergistic mechanisms with other cytotoxic molecules.

**Figure 6:**
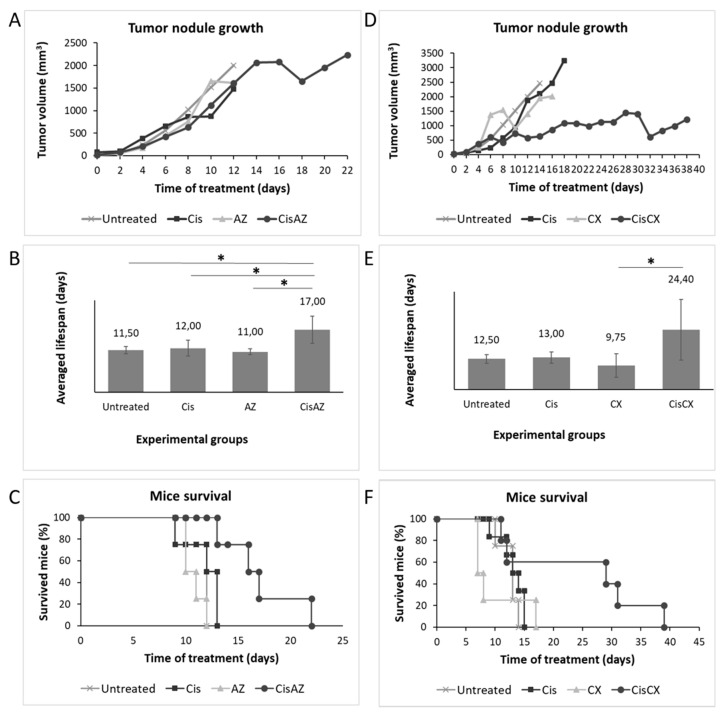
Pharmacological inhibition of MCM2 and CA9 in vivo. The effects of MCM2 and CA9 pharmacological inhibition on tumor nodule growth (**A**,**D**), averaged lifespan (**B**,**E**) and Kaplan Meier survival analysis (**C**,**F**) during the treatments with Acetazolamide and Ciprofloxacin are reposted as the average of each group of treatment. Significance in lifespans was calculated by Log rank test. Statistical significance is displayed as *p* < 0.05 (*).

In the first experiment, mice composing group 1 (*n* = 7) were subcutaneously injected with SKNBE2 cells, before receiving both acetazolamide (20mg/kg/dose, twice a day) and cisplatin (5 mg/kg/dose, once a week); a second group of mice was treated with cisplatin alone (5 mg/kg/dose), the third with acetazolamide alone (20 mg/kg/dose), whereas control group was treated with vehicle (saline solution). As shown in Figure 6A, mice treated with acetazolamide or with vehicle rapidly developed tumors, reaching the established maximal tumor mass threshold of 2.2 cm^3^ after 10 and 12 days, respectively, thus, did not show significant differences neither in tumor mass growth (0.22 cm^3^/die and 0.18 cm^3^/die respectively) nor in progression-free survival (PFS) (11 and 11.5 days for acetazolamide-treated and vehicle-treated mice, respectively; *p* = 0.27 Log rank test) (Figure 6B,C) confirming that acetazolamide in these experimental conditions does not exert an efficacious antitumor effect as a single agent (Figure 6A). Similarly, in these conditions, cisplatin-treated mice reached the tumor mass threshold after about the same time of control mice (the PFS of this group was 12 days; Log rank test *p* = 0.18 and 0.33 with respect to acetazolamide and control mice, respectively) (Figure 6B). On the contrary, the combined administration of cisplatin and acetazolamide reached the established tumor mass threshold of 2.2 cm^3^ after 22 days, corresponding to a tumor growth rate delayed to 0.1 cm^3^/die and a strongly improved PFS (22 days; *p* < 0.05, *p* < 0.01 and *p* = 0.01 Log rank test with respect to cisplatin, acetazolamide, and untreated control group, respectively). Altogether, these results demonstrate that the co-administration of acetazolamide was able to enhance significantly cisplatin efficacy in a combined therapy.

In the second experiment, we administered ciprofloxacin (70 mg/kg/dose, twice a day) together with cisplatin (5 mg/kg/dose, once a week). In this experiment control groups were cisplatin alone (5 mg/kg/dose), ciprofloxacin alone (70 mg/kg/dose), and vehicle (saline solution).

In both control groups, ciprofloxacin-treated and vehicle-treated mice, the nodule growth was rapid confirming that ciprofloxacin does not have an antitumor effect in NB as a single agent. In contrast, the group of mice treated with cisplatin /ciprofloxacin on average did not reach the maximum tumor volume thus the last individual of the group was sacrificed after 38 days (Figure 6D).

In the combinational treatment we observed a PFS of 24.40 days versus 13 days observed in cisplatin-treated mice (Log Rank Test, *p* = 0.14). Ciprofloxacin alone showed a PFS similar to that observed in the vehicle control group (9.75 days versus 12.50, respectively) (Figure 6D–F). Although, most likely due to the small number of animal treated, the Log Rank test performed on Kaplan Meier data yielded statistically significant differences only between the co-administration of cisplatin and ciprofloxacin if compared to ciprofloxacin alone control group (Log rank test, *p* < 0.05), the average lifespan of 24.4 days of cisplatin/ciprofloxacin-treated mice compared to that of 13 days shown by the only cisplatin-treated group is encouraging and again keeps in line with a key role of MCM2 played in NB aggressiveness (Figure 6E,F).

## 4. Discussion

Among significant advances in therapy of different cancer types, HR-NB still represents a major challenge still showing a very low survival rate. Thus, novel molecules are needed to develop more efficacious therapies. To this aim is of capital importance the availability of preclinical models of the disease that recapitulate the different stages of cancer cell transformation to obtain novel information. In this scenario we report an in vitro model endowed with the different intermediate stages of differentiation that span from the fully malignant Myc-N amplified to the fully mature neuron-like cells able to generate spontaneously a neural network.

This model, encompassing several intermediate NB differentiation stages, allows the selection of proteins specifically overexpressed at the two extreme points, fully malignant and non-malignant/fully differentiated, thus identifying only genes that are progressively overexpressed during the changes of phenotype. The restricted number of genes identified is reliable as demonstrated by validation in NB survival analysis that showed MCM2 as a novel element with very high prognostic value. Indeed, one of the main findings of this study was the identification of the ability of MCM2 to stratify NB patients on the basis of survival rates. In this scenario, the association of low survival with high expression of MCM2, further strengthened by the present in vivo experiments, suggests a pivotal oncogenic activity of this gene in NB.

In addition to single gene analysis, also gene cluster analysis reveals a signature for low survival prognosis thus strengthening the validity of this model. The analysis showed the association between these genes, age at diagnosis, and MYCN amplification. Notably, three genes overexpressed in differentiated cells and strongly down-regulated in the malignant stage (BIRC3, ERCC5, and LIG4) are key components of the DNA damage repair system, indicating that the malignant stage of NB cells is characterized by a constitutive genomic instability, which favoring the accumulation of novel mutations might, in turn, drive further stages of malignant transformation. The high expression of DDIT gene in Mock cells, a condition specifically induced by the accumulation of DNA damages, is in agreement with this view.

Gene interaction network analysis also provided, showing an intriguing overlap between the PTGs and PNGs genes with the TIN-signature genes [26], confirms the involvement of a diffused transcriptomic deregulation and a replication stress response associated with more aggressive phenotypes in this in vitro model as shown in NB tumors. Therefore, the set of genes that we have identified here represents a list of possible novel targets for innovative therapies. Thus, the availability of a series of inhibitors for these molecules which activity is particularly high in aggressive NB cells is of remarkable importance.

The prognostic value of MCM2 gene together with the wide interest for CA9 inhibitors, prompted us to focus on these two genes and their expression level in a heterotopic model of neuroblastomagenesis and in its treatment with anticancer therapy. Our results confirmed that these two genes are overexpressed in cancer nodules and that their expression is directly correlated with tumor mass. Notably, the correlation between the overexpression of these two genes and tumor growth was shown to be stronger than that derived from Ki67 labeling, commonly used as proliferation marker in tumor sections. Thus, we hypothesize that MCM2 and CA9 might be a valuable alternative to Ki67 to evaluate NB nodule growth in patients. Similarly, the correlation of the nodule growth rate and the expression of GD2 (commonly considered a marker of NB aggressiveness) is remarkably lower than that of MCM2 and CA9 on the same parameter, thus suggesting the use of these novel markers in the determination of the aggressiveness in NB nodules from patients.

In addition to a further validation of the reliability of our NB model, these results suggest the pharmacological inhibition of the activity of these gene products as promising novel anticancer therapy. The data here reported demonstrate the suitability of this hypothesis since ciprofloxacin and acetazolamide, inhibitors of MCM2 and CA9 activity, respectively, strongly potentiate anticancer effects of cisplatin in a heterotopic mouse model of NB. Acetazolamide is a reversible inhibitor of the carbonic anhydrase enzyme mainly used for glaucoma, and mountain sickness [43]. CA9 is expressed at low level only in few normal tissues, whereas it is over-expressed in several solid tumors in which, as a consequence of tumor hypoxic environment, converts CO_2_ to HCO_3_^−^ and H^+^. This expression profile makes CA9 a possible suitable selective target for novel therapeutic approaches. In fact, while we acknowledge that acetazolamide is a rather non-selective inhibitor of the different human carbonic anhydrase subtypes, novel more specific CA9 isoenzyme inhibitors could represent promising anticancer drug, in a drug repurposing perspective [44,45]. In this context our experiments strongly suggest its use also for the treatment of NB.

Mini-chromosome maintenance protein family (MCM) is involved in the initiation of eukaryotic genome replication [46]. In particular, MCM2 was reported by different studies to be a possible cancer marker [47,48,49]. Ciprofloxacin, a widely used antibiotic, inhibits MCM2 activity blocking cell cycle progression in S phase [50], but it is at the same time a MCM2-7 inhibitor [42,43,44,45,46,47,48,49,50,51]. The results we here report, strongly support the need of further preclinical studies aimed to assess the possible repositioning of ciprofloxacin in NB therapy.

## 5. Conclusions

Our work demonstrates that the NDM29-dependent differentiation model recapitulates the multistage process of NB tumorigenesis/differentiation and provides reliable information about novel target genes to be considered possible targets for NB therapy.

## Figures and Tables

**Table 1:** Survival analysis correlating PTGs and PNGs to NB aggressiveness.

	RNAseq498			Affymetrix88		
Covariate	HR	95% CI	*p* Value	HR	95% CI	*p* Value
MCM2	3.15	(2.51–3.96)	<2 × 10^−16^	2.29	(1.61–3.27)	4.07 × 10^−6^
FGF2	2.19	(1.63–2.93)	1.47 × 10^−7^	0.85	(0.59–1.23)	4.00 × 10^−1^
CA9	1.85	(1.37–2.49)	4.57 × 10^−5^	2.28	(1.24–4.2)	7.00 × 10^−3^
ADM	1.6	(1.38–1.97)	3.06 × 10^−8^	1.36	(0.89–2.06)	1.40 × 10^−1^
SERPINF1	1.44	(1.24–1.68)	2.56 × 10^−6^	1.49	(1.13–1.97)	4.00 × 10^−3^
TINF2	1.4	(0.75–2.59)	2.80 × 10^−1^	1.58	(0.53–4.74)	4.00 × 10^−1^
SNAI1	1.27	(0.99–1.64)	5.00 × 10^−2^	1.16	(0.86–1.55)	3.20 × 10^−1^
BMI1	1.17	(0.71–1.92)	5.00 × 10^−1^	0.44	(0.19–1.01)	5.00 × 10^−2^
ANGPT1	0.97	(0.77–1.24)	8.00 × 10^−1^	1.08	(0.69–1.69)	7.30 × 10^−1^
DDIT3	0.78	(0.53–1.15)	2.00 × 10^−1^	0.77	(0.45–1.33)	3.50 × 10^−1^
LIG4	0.74	(0.53–1.05)	9.00 × 10^−2^	0.73	(0.43–1.22)	2.40 × 10^−1^
STMN1	0.69	(0.51–0.94)	1.00 × 10^−2^	0.82	(0.46–1.45)	5.00 × 10^−1^
CCL2	0.68	(0.59–0.78)	1.82 × 10^−7^	1	(0.77–1.31)	9.40 × 10^−1^
BIRC3	0.65	(0.57–0.75)	6.41 × 10^−10^	0.88	(0.72–1.08)	2.40 × 10^−1^
TEK	0.61	(0.51–0.73)	4.99 × 10^−8^	0.77	(0.49–1.21)	2.70 × 10^−1^
SOX10	0.6	(0.51–0.70)	6.74 × 10^−10^	0.75	(0.60–0.94)	1.00 × 10^−2^
OCLN	0.56	(0.46–0. 68)	7.81 × 10^−9^	0.79	(0.65–0.96)	2.00 × 10^−2^
ACSL4	0.52	(0.37–0.73)	1.70 × 10^−4^	0.44	(0.23–0.82)	1.00 × 10^−2^
XIAP	0.46	(0.25–0.84)	1.00 × 10^−2^	0.21	(0.09–0.49)	2.00 × 10^−4^
ERCC5	0.23	(0.13–0.38)	1.88 × 10^−8^	0.36	(0.13–0.95)	4.00 × 10^−2^

Univariate analysis was assessed by Cox regression. Overall survival defined the time to event. Covariates are sorted by decreasing order of HR in the RNAseq498 dataset. HR = Hazard ratio. CI = Confidence interval.

## Supplementary Materials

The following are available online at https://www.mdpi.com/2227-9059/8/11/471/s1, Material Online 1: Table list of the primers used in real-time RT-PCR; Supplementary Material Online 2: Real time RT-PCR reaction panels validating results obtained using low density PCR arrays; Supplementary Material Online 3: Pearson’s correlation analysis of tumor nodules growth, dangerousness and MCM2/CA9 expression level.
