# Supplementary material for: MCM2 and Carbonic Anhydrase 9 Are Novel Potential Targets for Neuroblastoma Pharmacological Treatment
Source: Biomedicines. 2020 Nov 3;8(11):471. doi: 10.3390/biomedicines8110471 (PMC7692293; doi:10.3390/biomedicines8110471)
Supplement: Supplementary file 1 [file biomedicines-08-00471-s001.zip › Supplementary 1.pdf]

Gene	Forward	Reverse
**ACSL4**	GTC TCC TAT CTG ATT ACC AGT GTT GA	GTC CAC ATA AAT GAT ATG TTT AAC ACA ACT
**BIRC3**	ATC TGG AGA TGA TCC ATG GG	TGT TCA AGT AGA TGA GGG
**BMI1**	AAA TTC GGA GAC CTG GAA CAA G	GTT GTT CGA TGC ATT TCT GCT T
**ERCC5**	GAC AAG CCA TCA AAA CTG CCT	AAC TTG GGT AAG ACT GGG TAG T
**LIG4**	GCT GGG ATT CTC TGG TTC ACA	AGC GGT GAT GAA TCT TCT CGT T
**XIAP**	ACA AGG AGC AGC TTG CAA GAG	TCC TCC ACA GTG AAA GCA CTT TAC
**OCLN**	TCT CCC GTT TGG ATA AAG AAT TG	CAT CAG CAG CAG CCA TGT ACT C
**TEK**	GCT TGC TCC TTT CTG GAA CTG T	CGC CAC CCA GAG GCA AT
**CCL2**	CCC AGT CAC CTG CTG TTA	TGC TGC TGG TGA TTC TTC
**FGF2**	TGG CTA TGA AGG AAG ATG GAA GA	CAA TCG TTC AAA GAA ACA CTC A
**SOX10**	GCC CCT ATT AAC TCT GCA TTA AGC	CCC CAT TCA CCA AGC AGG TA
**DDIT3**	CTGGGGAATGACCACTCTGT	CCTTTCTCCTTCGGGACACT
**CA9**	AGG GTG TCA TCT GGA CTG TGT TT	GGG TGT GGA GCT GCT TAG CA
**ADM**	GGG CGC AAG CCT CAC TAT TA	CGC ATT GCA CTT TTC CTC TTT
**SERPINF1**	TGA AGG CGA AGT CAC CAA GTC	GAT CTT GCT AAA GTC TGG TGA ATC AA
**ANGPT1**	GCA ACT GGA GCT GAT GGA CAC A	CAT CTG CAC AGT CTC TAA ATG GT
**MCM2**	CAC ACA GAA GTT CAG CGT CAT G	CAC GCC GGA ARG AAA GGT A
**SNAI1**	CAC TAT GCC GCG CTC TTC	GGT CGT AGG GCT GCT GGA A
**STMN1**	TAG AAT CTT GAG ATT CTC TCT C	CAG GTC AGC TGT TAC TTT
**TINF2**	CTC TTG CCT GGA GAC AAT ATG GT	GGC TCA GCC AGG TTC ACT GA
**GAPDH**	GAA GAT GGT GAT GGG ATT TC	GAA GGT GAA GGT CGG AGT C
**29A**	GCT TGT TGA AGG CTT TGA AGG CTT TGA TCT TA	GGG ATT ACA CAC AGG AAG CCA
