# Supplementary material for: MCM2 and Carbonic Anhydrase 9 Are Novel Potential Targets for Neuroblastoma Pharmacological Treatment
Source: Biomedicines. 2020 Nov 3;8(11):471. doi: 10.3390/biomedicines8110471 (PMC7692293; doi:10.3390/biomedicines8110471)
Supplement: Supplementary file 1 [file biomedicines-08-00471-s001.zip › Supplementary 3.pdf]

**Untreated**	**Pearson**	**Correlation**
Tunel	-0.757	Neg. Strong
Ki67	0.39	Moderate
GD2	0.5301	Moderate
MCM2	0.8391	Strong
CA9	0.8841	Strong

**Cisplatin-treated**	**Pearson**	**Correlation**
Tunel	-0.705	Neg. strong
Ki67	0.53	Moderate
GD2	0.6735	Moderate
MCM2	0.8557	Strong
CA9	0.901	Strong
