# Supplementary figures and images for: MCM2 and Carbonic Anhydrase 9 Are Novel Potential Targets for Neuroblastoma Pharmacological Treatment
Source: Biomedicines. 2020 Nov 3;8(11):471. doi: 10.3390/biomedicines8110471 (PMC7692293; doi:10.3390/biomedicines8110471)

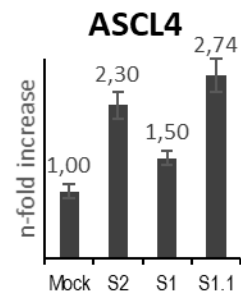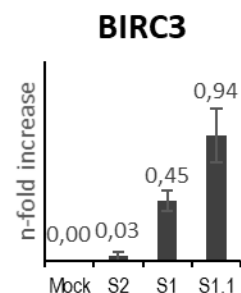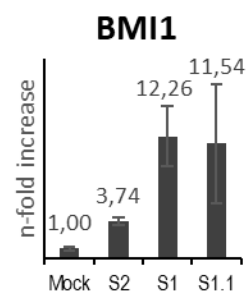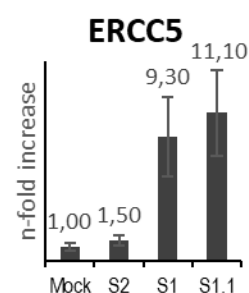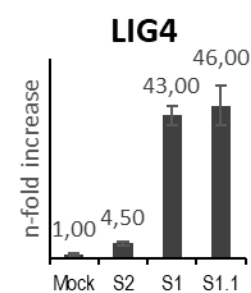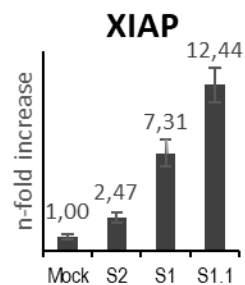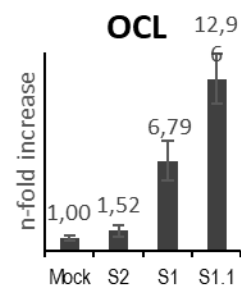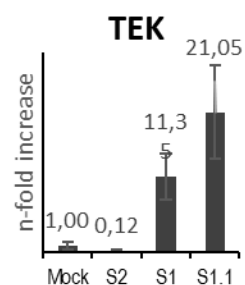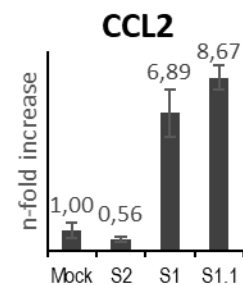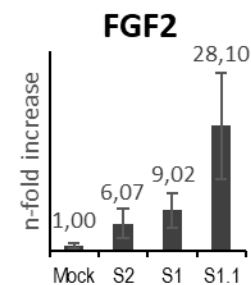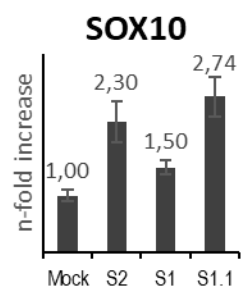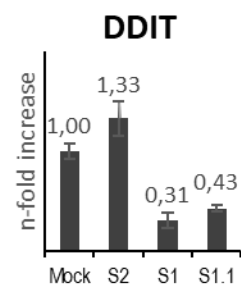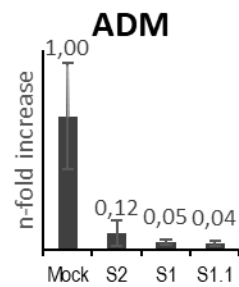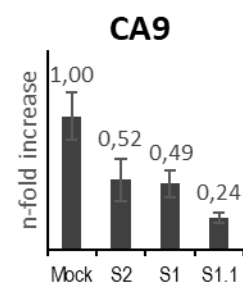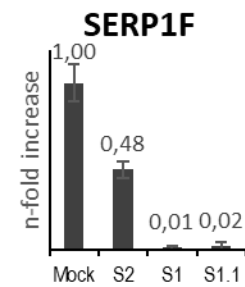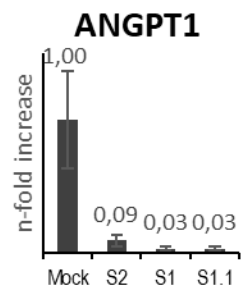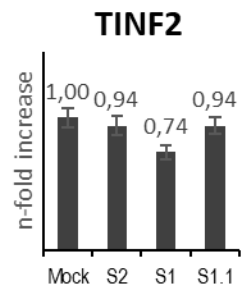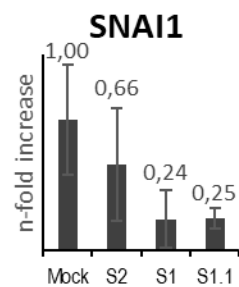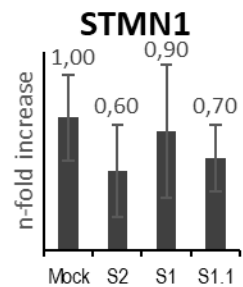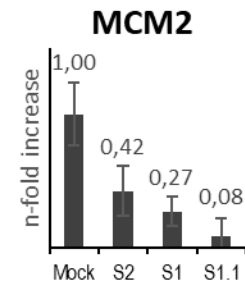

Supplement: Supplementary file 1 [file biomedicines-08-00471-s001.zip › Supplementary 2.pdf]
